# Crystal structure of 7-isopropyl-1,4a,*N*-trimethyl-1,2,3,4,4a,4b,5,6,7,8,10,10a-dodeca­hydro­phenanthrene-1-carb­ox­amide

**DOI:** 10.1107/S2056989015017648

**Published:** 2015-09-26

**Authors:** Li Liu, Xin-Yan Yan, Xiao-Ping Rao

**Affiliations:** a Institute of Chemical Industry of Forest Products, Chinese Academy of Forestry, Key Laboratory of Biomass Energy and Material, Jiangsu Province, National Engineering Lab. for Biomass Chemical Utilization, Key and Lab. on Forest Chemical Engineering, SFA, Nanjing, 210042, People’s Republic of China

**Keywords:** crystal structure, di­hydro­abietic acid derivative, C—H⋯π inter­actions

## Abstract

In the title compound, C_26_H_37_NO, a new derivative of di­hydro­abietic acid, the two cyclo­hexene rings adopt half chair conformations, whereas the cyclo­hexane ring has a chair conformation. Each of the methyl groups is in an axial position with respect to the tricyclic hydro­phenanthrene residue. In the crystal packing, methyl­ene-C—H⋯π(phen­yl) inter­actions lead to supra­molecular helical chains along [010]; the amide-H atom does not form a significant inter­molecular inter­action owing to steric pressure.

## Related literature   

For crystal structure of di­hydro­abietic acid derivatives, see: Rao *et al.* (2009 ▸); Rao (2010 ▸). For the biological activity of rosin acid derivatives, see: Fonseca *et al.* (2004 ▸); Gonzaléz *et al.* (2010 ▸); Rao *et al.* (2008 ▸); Sepulveda *et al.* (2005 ▸); Xing *et al.* (2013 ▸).
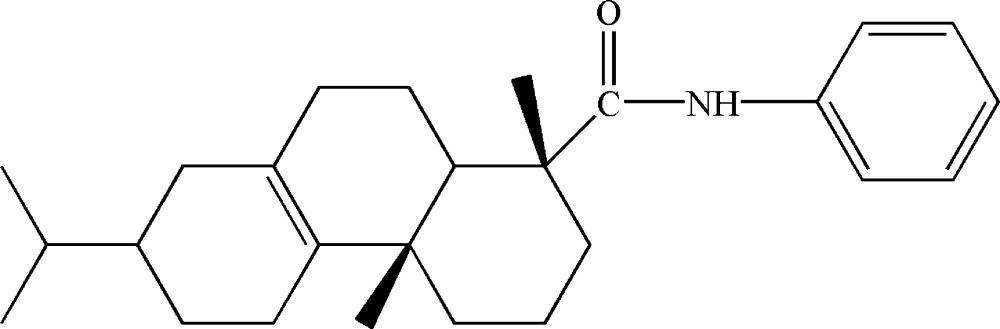


## Experimental   

### Crystal data  

C_26_H_37_NO
*M* *_r_* = 379.57
Orthorhombic, 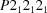

*a* = 26.223 (5) Å
*b* = 5.9230 (12) Å
*c* = 14.493 (3) Å
*V* = 2251.0 (8) Å^3^
*Z* = 4
Mo *K*α radiation
μ = 0.07 mm^−1^
*T* = 293 K
0.20 × 0.20 × 0.10 mm

### Data collection  

Enraf–Nonius CAD-4 diffractometer
Absorption correction: ψ scan (*CAD-4 Software*; Enraf–Nonius, 1985 ▸) *T*_min_ = 0.987, *T*_max_ = 0.993
4707 measured reflections
4122 independent reflections
2080 reflections with *I* > 2σ(*I*)
*R*_int_ = 0.099
3 standard reflections every 200 reflections intensity decay: 1%

### Refinement  

*R*[*F* ^2^ > 2σ(*F* ^2^)] = 0.079
*wR*(*F* ^2^) = 0.193
*S* = 1.01
4122 reflections
254 parameters
H-atom parameters constrained
Δρ_max_ = 0.21 e Å^−3^
Δρ_min_ = −0.30 e Å^−3^

### 

Data collection: *CAD-4 Software* (Enraf–Nonius, 1985 ▸); cell refinement: *CAD-4 Software*; data reduction: *XCAD4* (Harms & Wocadlo, 1995 ▸); program(s) used to solve structure: *SHELXS97* (Sheldrick, 2008 ▸); program(s) used to refine structure: *SHELXL97* (Sheldrick, 2008 ▸); molecular graphics: *ORTEP-3 for Windows* (Farrugia, 2012 ▸); software used to prepare material for publication: *SHELXL97*.

## Supplementary Material

Crystal structure: contains datablock(s) I, global. DOI: 10.1107/S2056989015017648/tk5389sup1.cif

Structure factors: contains datablock(s) I. DOI: 10.1107/S2056989015017648/tk5389Isup2.hkl

**Fig. 1.**
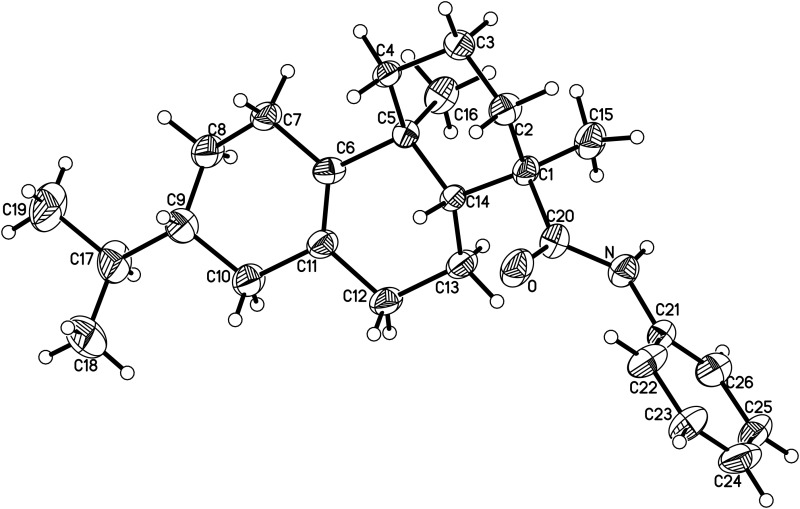
Molecular structure of the title compound, with H atoms represented by small spheres of arbitrary radius and displacement ellipsoids at the 30% probability level.

. DOI: 10.1107/S2056989015017648/tk5389fig1.tif
Mol­ecular structure of the title compound, with H atoms represented by small spheres of arbitrary radius and displacement ellipsoids at the 30% probability level.

CCDC reference: 1426243

Additional supporting information:  crystallographic information; 3D view; checkCIF report

## Figures and Tables

**Table 1:** Hydrogen-bond geometry (, ) *Cg*1 is the centroid of the C21C26 ring.

*D*H*A*	*D*H	H*A*	*D* *A*	*D*H*A*
C3H3*A* *Cg*1^i^	0.97	2.82	3.705(5)	151

Symmetry code: (i) 
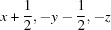
.

## supplementary crystallographic information

### S1. Comment

Rosin acid derivatives exhibit wide range of biological activities, such as antifungal and antitumor (Fonseca *et al.*, 2004; Rao *et al.*, 2008; Gonzaléz *et al.*, 2008; Xing *et al.*, 2013.) activities. Nitrogen derivatives of rosin acid have been studied as cytotoxic reagents and they are found to have high activity in reducing blood serum cholesterol levels in animals (Sepulveda *et al.*, 2005). In this work, we describe the crystal structure of the title compound.

Dihydroabietic acid is one of the main component of hydrogenated rosin, which can be isolated from hydrogenated rosin by recrystallization. In this work, we have obtained the single crystal structure of the title compound, the tricyclic hydrophenanthrene nuclei had the similar crystal structure with dihydroabietic acid derivatives (Rao *et al.*, 2009; Rao, 2010). The two cyclohexenes adopt half chair conformations, whereas the cyclohexane has a chair conformation (Fig. 1). The two methyl groups are in axial positions with respect to the tricyclic hydrophenanthrene nuclei. The structures of related dihydroabietic acid derivatives are known (Rao *et al.* 2009; Rao, 2010)

### S2. Experimental

A mixture of dihydroabietic acid (0.1 mol), oxalyl chloride (0.11 mol) and dichloromethane (40 ml) was stirred at 313 K for 4 h. After distilling off the solvent, the residue was added to aniline (0.2 mol) in toluene (60 ml) solution. The mixture was reacted for 24 h at room temperature. The solvent was distilled off, and upon recrystallization from acetone, white crystals of the title compound were obtained (yield 53%, M.p. 422 K). Single crystals were grown from acetone.

### S3. Refinement

H atoms were positioned geometrically and refined as riding atoms, with C—H = 0.96 Å and *U*_iso_(H) = 1.5*U*_eq_(C) for methyl H atoms, and C—H = 0.97–0.98 Å and *U*_iso_(H) = 1.2*U*_eq_(C) for all other H atoms. The absolute structure was not determined.

### Crystal data

**Table:**

C_26_H_37_NO	*D*_x_ = 1.120 Mg m^−^^3^
*M**_r_* = 379.57	Mo *K*α radiation, λ = 0.71073 Å
Orthorhombic, *P*2_1_2_1_2_1_	Cell parameters from 25 reflections
*a* = 26.223 (5) Å	θ = 9–13°
*b* = 5.9230 (12) Å	µ = 0.07 mm^−^^1^
*c* = 14.493 (3) Å	*T* = 293 K
*V* = 2251.0 (8) Å^3^	Block, white
*Z* = 4	0.20 × 0.20 × 0.10 mm
*F*(000) = 832	

### Data collection

**Table:**

Enraf–Nonius CAD-4 diffractometer	2080 reflections with *I* > 2σ(*I*)
Radiation source: fine-focus sealed tube	*R*_int_ = 0.099
Graphite monochromator	θ_max_ = 25.4°, θ_min_ = 1.6°
ω/2θ scans	*h* = −31→31
Absorption correction: ψ scan (*CAD-4 Software*; Enraf–Nonius, 1985)	*k* = 0→7
*T*_min_ = 0.987, *T*_max_ = 0.993	*l* = 0→17
4707 measured reflections	3 standard reflections every 200 reflections
4122 independent reflections	intensity decay: 1%

### Refinement

**Table:**

Refinement on *F*^2^	Secondary atom site location: difference Fourier map
Least-squares matrix: full	Hydrogen site location: inferred from neighbouring sites
*R*[*F*^2^ > 2σ(*F*^2^)] = 0.079	H-atom parameters constrained
*wR*(*F*^2^) = 0.193	*w* = 1/[σ^2^(*F*_o_^2^) + (0.040*P*)^2^ + 2.3*P*] where *P* = (*F*_o_^2^ + 2*F*_c_^2^)/3
*S* = 1.01	(Δ/σ)_max_ < 0.001
4122 reflections	Δρ_max_ = 0.21 e Å^−^^3^
254 parameters	Δρ_min_ = −0.30 e Å^−^^3^
0 restraints	Absolute structure: nd
Primary atom site location: structure-invariant direct methods	

### Special details

**Table:**

Geometry. All e.s.d.'s (except the e.s.d. in the dihedral angle between two l.s. planes) are estimated using the full covariance matrix. The cell e.s.d.'s are taken into account individually in the estimation of e.s.d.'s in distances, angles and torsion angles; correlations between e.s.d.'s in cell parameters are only used when they are defined by crystal symmetry. An approximate (isotropic) treatment of cell e.s.d.'s is used for estimating e.s.d.'s involving l.s. planes.
Refinement. Refinement of *F*^2^ against ALL reflections. The weighted *R*-factor *wR* and goodness of fit *S* are based on *F*^2^, conventional *R*-factors *R* are based on *F*, with *F* set to zero for negative *F*^2^. The threshold expression of *F*^2^ > σ(*F*^2^) is used only for calculating *R*-factors(gt) *etc*. and is not relevant to the choice of reflections for refinement. *R*-factors based on *F*^2^ are statistically about twice as large as those based on *F*, and *R*- factors based on ALL data will be even larger.

### Fractional atomic coordinates and isotropic or equivalent isotropic displacement parameters (Å^2^)

**Table:**

	*x*	*y*	*z*	*U*_iso_*/*U*_eq_	
N	0.04603 (15)	0.2213 (8)	0.1116 (3)	0.0541 (12)	
H0A	0.0340	0.3561	0.1081	0.065*	
O	0.02279 (14)	−0.1458 (7)	0.1135 (3)	0.0712 (12)	
C1	−0.04586 (18)	0.1222 (9)	0.0953 (3)	0.0443 (13)	
C2	−0.07609 (18)	0.0042 (11)	0.1719 (3)	0.0547 (15)	
H2A	−0.0656	−0.1526	0.1756	0.066*	
H2B	−0.0685	0.0752	0.2306	0.066*	
C3	−0.13357 (19)	0.0159 (10)	0.1540 (3)	0.0534 (15)	
H3A	−0.1514	−0.0652	0.2025	0.064*	
H3B	−0.1445	0.1723	0.1562	0.064*	
C4	−0.14786 (18)	−0.0847 (9)	0.0608 (3)	0.0444 (13)	
H4A	−0.1844	−0.0719	0.0520	0.053*	
H4B	−0.1392	−0.2439	0.0603	0.053*	
C5	−0.12012 (18)	0.0350 (9)	−0.0201 (3)	0.0373 (12)	
C6	−0.12908 (18)	−0.1043 (9)	−0.1095 (3)	0.0444 (13)	
C7	−0.18354 (18)	−0.1681 (10)	−0.1295 (3)	0.0515 (15)	
H7A	−0.2058	−0.0466	−0.1096	0.062*	
H7B	−0.1922	−0.3016	−0.0941	0.062*	
C8	−0.1931 (2)	−0.2149 (12)	−0.2312 (4)	0.0659 (18)	
H8A	−0.2269	−0.2785	−0.2388	0.079*	
H8B	−0.1917	−0.0742	−0.2653	0.079*	
C9	−0.1547 (2)	−0.3739 (12)	−0.2690 (3)	0.0620 (17)	
H9A	−0.1558	−0.5079	−0.2294	0.074*	
C10	−0.1015 (2)	−0.2759 (12)	−0.2579 (4)	0.0637 (17)	
H10A	−0.0770	−0.3989	−0.2588	0.076*	
H10B	−0.0943	−0.1800	−0.3106	0.076*	
C11	−0.0933 (2)	−0.1404 (10)	−0.1712 (4)	0.0521 (15)	
C12	−0.03866 (19)	−0.0583 (12)	−0.1611 (3)	0.0645 (18)	
H12A	−0.0165	−0.1873	−0.1512	0.077*	
H12B	−0.0282	0.0143	−0.2180	0.077*	
C13	−0.03203 (18)	0.1058 (11)	−0.0822 (3)	0.0561 (16)	
H13A	−0.0443	0.2538	−0.1003	0.067*	
H13B	0.0038	0.1190	−0.0668	0.067*	
C14	−0.06178 (17)	0.0224 (9)	0.0016 (3)	0.0416 (13)	
H14A	−0.0541	−0.1391	0.0060	0.050*	
C15	−0.0514 (2)	0.3815 (10)	0.1043 (4)	0.0629 (16)	
H15A	−0.0868	0.4197	0.1110	0.094*	
H15B	−0.0380	0.4529	0.0500	0.094*	
H15C	−0.0329	0.4328	0.1575	0.094*	
C16	−0.1422 (2)	0.2680 (10)	−0.0338 (4)	0.0597 (16)	
H16A	−0.1367	0.3567	0.0207	0.089*	
H16B	−0.1782	0.2562	−0.0454	0.089*	
H16C	−0.1259	0.3393	−0.0854	0.089*	
C17	−0.1645 (3)	−0.4587 (13)	−0.3682 (4)	0.081 (2)	
H17A	−0.1606	−0.3274	−0.4087	0.097*	
C18	−0.1258 (3)	−0.6326 (14)	−0.4005 (4)	0.109 (3)	
H18A	−0.1333	−0.6755	−0.4629	0.164*	
H18B	−0.1275	−0.7632	−0.3614	0.164*	
H18C	−0.0922	−0.5688	−0.3977	0.164*	
C19	−0.2188 (3)	−0.5441 (16)	−0.3825 (4)	0.114 (3)	
H19A	−0.2230	−0.5928	−0.4452	0.170*	
H19B	−0.2425	−0.4247	−0.3696	0.170*	
H19C	−0.2252	−0.6687	−0.3417	0.170*	
C20	0.0111 (2)	0.0508 (10)	0.1082 (4)	0.0501 (14)	
C21	0.09969 (19)	0.2039 (9)	0.1201 (4)	0.0455 (13)	
C22	0.1229 (2)	0.0189 (13)	0.1586 (4)	0.0669 (18)	
H22A	0.1035	−0.1027	0.1788	0.080*	
C23	0.1762 (2)	0.0137 (13)	0.1673 (4)	0.074 (2)	
H23A	0.1921	−0.1103	0.1940	0.089*	
C24	0.2042 (2)	0.1900 (14)	0.1367 (4)	0.082 (2)	
H24A	0.2395	0.1847	0.1415	0.098*	
C25	0.1812 (2)	0.3788 (12)	0.0981 (4)	0.0692 (18)	
H25A	0.2005	0.5010	0.0781	0.083*	
C26	0.1281 (2)	0.3796 (11)	0.0904 (4)	0.0614 (16)	
H26A	0.1119	0.5038	0.0642	0.074*	

### Atomic displacement parameters (Å^2^)

**Table:**

	*U*^11^	*U*^22^	*U*^33^	*U*^12^	*U*^13^	*U*^23^
N	0.045 (3)	0.058 (3)	0.060 (3)	−0.004 (2)	−0.007 (2)	0.003 (3)
O	0.046 (2)	0.066 (3)	0.101 (3)	0.001 (2)	−0.021 (2)	−0.003 (3)
C1	0.035 (3)	0.054 (3)	0.044 (3)	−0.005 (3)	−0.005 (2)	−0.003 (3)
C2	0.053 (4)	0.071 (4)	0.040 (3)	−0.005 (3)	−0.006 (3)	0.002 (3)
C3	0.053 (3)	0.068 (4)	0.040 (3)	−0.008 (3)	0.006 (2)	−0.008 (3)
C4	0.035 (3)	0.054 (3)	0.044 (3)	−0.007 (3)	0.004 (2)	0.001 (3)
C5	0.039 (3)	0.041 (3)	0.032 (3)	−0.005 (2)	−0.001 (2)	0.002 (2)
C6	0.036 (3)	0.060 (3)	0.037 (3)	0.003 (3)	0.000 (2)	0.006 (3)
C7	0.037 (3)	0.071 (4)	0.047 (3)	0.000 (3)	−0.004 (2)	−0.007 (3)
C8	0.045 (4)	0.103 (5)	0.049 (4)	−0.007 (4)	−0.009 (3)	−0.010 (4)
C9	0.070 (4)	0.093 (5)	0.024 (3)	−0.015 (4)	−0.004 (3)	−0.004 (3)
C10	0.052 (4)	0.098 (5)	0.041 (3)	−0.009 (4)	−0.004 (3)	−0.012 (3)
C11	0.042 (3)	0.069 (4)	0.046 (3)	−0.005 (3)	−0.002 (3)	0.000 (3)
C12	0.041 (3)	0.111 (5)	0.041 (3)	−0.017 (4)	0.005 (3)	−0.012 (4)
C13	0.038 (3)	0.083 (4)	0.047 (3)	−0.011 (3)	0.002 (2)	−0.005 (3)
C14	0.034 (3)	0.049 (3)	0.042 (3)	−0.006 (2)	0.001 (2)	−0.003 (3)
C15	0.055 (4)	0.062 (4)	0.072 (4)	−0.005 (3)	−0.017 (3)	−0.003 (3)
C16	0.053 (4)	0.062 (4)	0.064 (4)	0.001 (3)	−0.016 (3)	0.010 (3)
C17	0.097 (5)	0.108 (6)	0.038 (3)	−0.031 (5)	−0.006 (3)	−0.007 (4)
C18	0.131 (7)	0.141 (8)	0.055 (4)	−0.017 (6)	0.018 (5)	−0.035 (5)
C19	0.102 (6)	0.178 (9)	0.061 (4)	−0.048 (6)	−0.022 (4)	−0.022 (6)
C20	0.049 (3)	0.049 (3)	0.052 (3)	−0.004 (3)	−0.009 (3)	0.009 (3)
C21	0.039 (3)	0.050 (3)	0.047 (3)	−0.004 (3)	−0.002 (3)	0.003 (3)
C22	0.039 (4)	0.100 (5)	0.062 (4)	−0.009 (4)	−0.002 (3)	0.018 (4)
C23	0.041 (4)	0.093 (5)	0.087 (5)	−0.005 (4)	−0.009 (3)	0.015 (4)
C24	0.041 (4)	0.135 (7)	0.069 (5)	0.002 (4)	0.002 (3)	−0.005 (5)
C25	0.050 (4)	0.095 (5)	0.063 (4)	−0.030 (4)	0.005 (3)	0.008 (4)
C26	0.055 (4)	0.082 (5)	0.047 (3)	−0.007 (3)	−0.005 (3)	0.009 (3)

### Geometric parameters (Å, º)

**Table:**

N—C20	1.364 (6)	C11—C12	1.519 (7)
N—C21	1.416 (6)	C12—C13	1.511 (7)
N—H0A	0.8600	C12—H12A	0.9700
O—C20	1.206 (6)	C12—H12B	0.9700
C1—C2	1.532 (7)	C13—C14	1.526 (6)
C1—C14	1.539 (6)	C13—H13A	0.9700
C1—C15	1.548 (7)	C13—H13B	0.9700
C1—C20	1.564 (7)	C14—H14A	0.9800
C2—C3	1.531 (6)	C15—H15A	0.9600
C2—H2A	0.9700	C15—H15B	0.9600
C2—H2B	0.9700	C15—H15C	0.9600
C3—C4	1.524 (6)	C16—H16A	0.9600
C3—H3A	0.9700	C16—H16B	0.9600
C3—H3B	0.9700	C16—H16C	0.9600
C4—C5	1.551 (6)	C17—C18	1.520 (9)
C4—H4A	0.9700	C17—C19	1.525 (8)
C4—H4B	0.9700	C17—H17A	0.9800
C5—C16	1.510 (7)	C18—H18A	0.9600
C5—C6	1.555 (6)	C18—H18B	0.9600
C5—C14	1.564 (6)	C18—H18C	0.9600
C6—C11	1.315 (6)	C19—H19A	0.9600
C6—C7	1.505 (6)	C19—H19B	0.9600
C7—C8	1.520 (7)	C19—H19C	0.9600
C7—H7A	0.9700	C21—C26	1.350 (7)
C7—H7B	0.9700	C21—C22	1.372 (7)
C8—C9	1.484 (8)	C22—C23	1.402 (7)
C8—H8A	0.9700	C22—H22A	0.9300
C8—H8B	0.9700	C23—C24	1.351 (9)
C9—C10	1.519 (7)	C23—H23A	0.9300
C9—C17	1.544 (7)	C24—C25	1.388 (9)
C9—H9A	0.9800	C24—H24A	0.9300
C10—C11	1.506 (7)	C25—C26	1.397 (7)
C10—H10A	0.9700	C25—H25A	0.9300
C10—H10B	0.9700	C26—H26A	0.9300
			
C20—N—C21	128.0 (5)	C13—C12—H12B	109.0
C20—N—H0A	116.0	C11—C12—H12B	109.0
C21—N—H0A	116.0	H12A—C12—H12B	107.8
C2—C1—C14	108.9 (4)	C12—C13—C14	109.6 (4)
C2—C1—C15	110.1 (5)	C12—C13—H13A	109.8
C14—C1—C15	115.4 (5)	C14—C13—H13A	109.8
C2—C1—C20	106.6 (4)	C12—C13—H13B	109.8
C14—C1—C20	105.1 (4)	C14—C13—H13B	109.8
C15—C1—C20	110.4 (4)	H13A—C13—H13B	108.2
C3—C2—C1	111.5 (4)	C13—C14—C1	116.1 (4)
C3—C2—H2A	109.3	C13—C14—C5	109.0 (4)
C1—C2—H2A	109.3	C1—C14—C5	115.1 (4)
C3—C2—H2B	109.3	C13—C14—H14A	105.2
C1—C2—H2B	109.3	C1—C14—H14A	105.2
H2A—C2—H2B	108.0	C5—C14—H14A	105.2
C4—C3—C2	112.0 (4)	C1—C15—H15A	109.5
C4—C3—H3A	109.2	C1—C15—H15B	109.5
C2—C3—H3A	109.2	H15A—C15—H15B	109.5
C4—C3—H3B	109.2	C1—C15—H15C	109.5
C2—C3—H3B	109.2	H15A—C15—H15C	109.5
H3A—C3—H3B	107.9	H15B—C15—H15C	109.5
C3—C4—C5	112.1 (4)	C5—C16—H16A	109.5
C3—C4—H4A	109.2	C5—C16—H16B	109.5
C5—C4—H4A	109.2	H16A—C16—H16B	109.5
C3—C4—H4B	109.2	C5—C16—H16C	109.5
C5—C4—H4B	109.2	H16A—C16—H16C	109.5
H4A—C4—H4B	107.9	H16B—C16—H16C	109.5
C16—C5—C4	109.7 (4)	C18—C17—C19	110.9 (6)
C16—C5—C6	108.5 (4)	C18—C17—C9	113.3 (6)
C4—C5—C6	108.5 (4)	C19—C17—C9	113.0 (5)
C16—C5—C14	116.5 (4)	C18—C17—H17A	106.3
C4—C5—C14	106.6 (4)	C19—C17—H17A	106.3
C6—C5—C14	106.9 (4)	C9—C17—H17A	106.3
C11—C6—C7	120.4 (5)	C17—C18—H18A	109.5
C11—C6—C5	123.1 (5)	C17—C18—H18B	109.5
C7—C6—C5	115.9 (4)	H18A—C18—H18B	109.5
C6—C7—C8	112.9 (4)	C17—C18—H18C	109.5
C6—C7—H7A	109.0	H18A—C18—H18C	109.5
C8—C7—H7A	109.0	H18B—C18—H18C	109.5
C6—C7—H7B	109.0	C17—C19—H19A	109.5
C8—C7—H7B	109.0	C17—C19—H19B	109.5
H7A—C7—H7B	107.8	H19A—C19—H19B	109.5
C9—C8—C7	111.3 (5)	C17—C19—H19C	109.5
C9—C8—H8A	109.4	H19A—C19—H19C	109.5
C7—C8—H8A	109.4	H19B—C19—H19C	109.5
C9—C8—H8B	109.4	O—C20—N	122.8 (5)
C7—C8—H8B	109.4	O—C20—C1	120.7 (5)
H8A—C8—H8B	108.0	N—C20—C1	116.4 (5)
C8—C9—C10	109.9 (5)	C26—C21—C22	120.0 (5)
C8—C9—C17	115.9 (5)	C26—C21—N	117.6 (5)
C10—C9—C17	112.2 (5)	C22—C21—N	122.4 (5)
C8—C9—H9A	106.0	C21—C22—C23	119.8 (6)
C10—C9—H9A	106.0	C21—C22—H22A	120.1
C17—C9—H9A	106.0	C23—C22—H22A	120.1
C11—C10—C9	115.0 (4)	C24—C23—C22	119.7 (7)
C11—C10—H10A	108.5	C24—C23—H23A	120.2
C9—C10—H10A	108.5	C22—C23—H23A	120.2
C11—C10—H10B	108.5	C23—C24—C25	121.2 (6)
C9—C10—H10B	108.5	C23—C24—H24A	119.4
H10A—C10—H10B	107.5	C25—C24—H24A	119.4
C6—C11—C10	123.5 (5)	C24—C25—C26	118.0 (6)
C6—C11—C12	123.8 (5)	C24—C25—H25A	121.0
C10—C11—C12	112.7 (4)	C26—C25—H25A	121.0
C13—C12—C11	112.8 (4)	C21—C26—C25	121.4 (6)
C13—C12—H12A	109.0	C21—C26—H26A	119.3
C11—C12—H12A	109.0	C25—C26—H26A	119.3
			
C14—C1—C2—C3	−54.0 (6)	C15—C1—C14—C13	60.9 (6)
C15—C1—C2—C3	73.4 (6)	C20—C1—C14—C13	−61.0 (6)
C20—C1—C2—C3	−166.9 (5)	C2—C1—C14—C5	56.2 (6)
C1—C2—C3—C4	56.7 (6)	C15—C1—C14—C5	−68.1 (6)
C2—C3—C4—C5	−58.1 (6)	C20—C1—C14—C5	170.0 (4)
C3—C4—C5—C16	−71.7 (5)	C16—C5—C14—C13	−65.6 (6)
C3—C4—C5—C6	169.9 (4)	C4—C5—C14—C13	171.6 (4)
C3—C4—C5—C14	55.2 (5)	C6—C5—C14—C13	55.8 (5)
C16—C5—C6—C11	101.6 (6)	C16—C5—C14—C1	66.8 (6)
C4—C5—C6—C11	−139.2 (5)	C4—C5—C14—C1	−56.0 (5)
C14—C5—C6—C11	−24.7 (7)	C6—C5—C14—C1	−171.8 (4)
C16—C5—C6—C7	−69.5 (6)	C8—C9—C17—C18	−176.2 (6)
C4—C5—C6—C7	49.6 (6)	C10—C9—C17—C18	56.4 (8)
C14—C5—C6—C7	164.2 (4)	C8—C9—C17—C19	−49.0 (9)
C11—C6—C7—C8	−14.9 (8)	C10—C9—C17—C19	−176.3 (7)
C5—C6—C7—C8	156.5 (5)	C21—N—C20—O	0.9 (9)
C6—C7—C8—C9	49.8 (7)	C21—N—C20—C1	−178.2 (5)
C7—C8—C9—C10	−59.3 (6)	C2—C1—C20—O	52.5 (7)
C7—C8—C9—C17	172.2 (5)	C14—C1—C20—O	−62.9 (7)
C8—C9—C10—C11	35.4 (7)	C15—C1—C20—O	172.0 (6)
C17—C9—C10—C11	165.9 (6)	C2—C1—C20—N	−128.3 (5)
C7—C6—C11—C10	−9.5 (9)	C14—C1—C20—N	116.2 (5)
C5—C6—C11—C10	179.7 (5)	C15—C1—C20—N	−8.8 (7)
C7—C6—C11—C12	173.0 (5)	C20—N—C21—C26	157.2 (6)
C5—C6—C11—C12	2.3 (9)	C20—N—C21—C22	−24.0 (9)
C9—C10—C11—C6	−1.1 (9)	C26—C21—C22—C23	0.4 (9)
C9—C10—C11—C12	176.7 (6)	N—C21—C22—C23	−178.4 (5)
C6—C11—C12—C13	−10.9 (8)	C21—C22—C23—C24	−0.8 (10)
C10—C11—C12—C13	171.4 (5)	C22—C23—C24—C25	1.2 (11)
C11—C12—C13—C14	42.7 (7)	C23—C24—C25—C26	−1.1 (10)
C12—C13—C14—C1	160.7 (4)	C22—C21—C26—C25	−0.3 (9)
C12—C13—C14—C5	−67.4 (6)	N—C21—C26—C25	178.5 (5)
C2—C1—C14—C13	−174.8 (5)	C24—C25—C26—C21	0.7 (9)

### Hydrogen-bond geometry (Å, º)

Cg1 is the centroid of the C21–C26 ring.

**Table:**

*D*—H···*A*	*D*—H	H···*A*	*D*···*A*	*D*—H···*A*
C3—H3*A*···*Cg*1^i^	0.97	2.82	3.705 (5)	151

Symmetry code: (i) *x*+1/2, −*y*−1/2, −*z*.
