# Supplementary figures and images for: Crystal structure of 7-isopropyl-1,4a,N-trimethyl-1,2,3,4,4a,4b,5,6,7,8,10,10a-dodeca­hydro­phenanthrene-1-carb­ox­amide
Source: Acta Crystallogr E Crystallogr Commun. 2015 Sep 26;71(Pt 10):o801–2. doi: 10.1107/S2056989015017648 (PMC4647402; doi:10.1107/S2056989015017648)

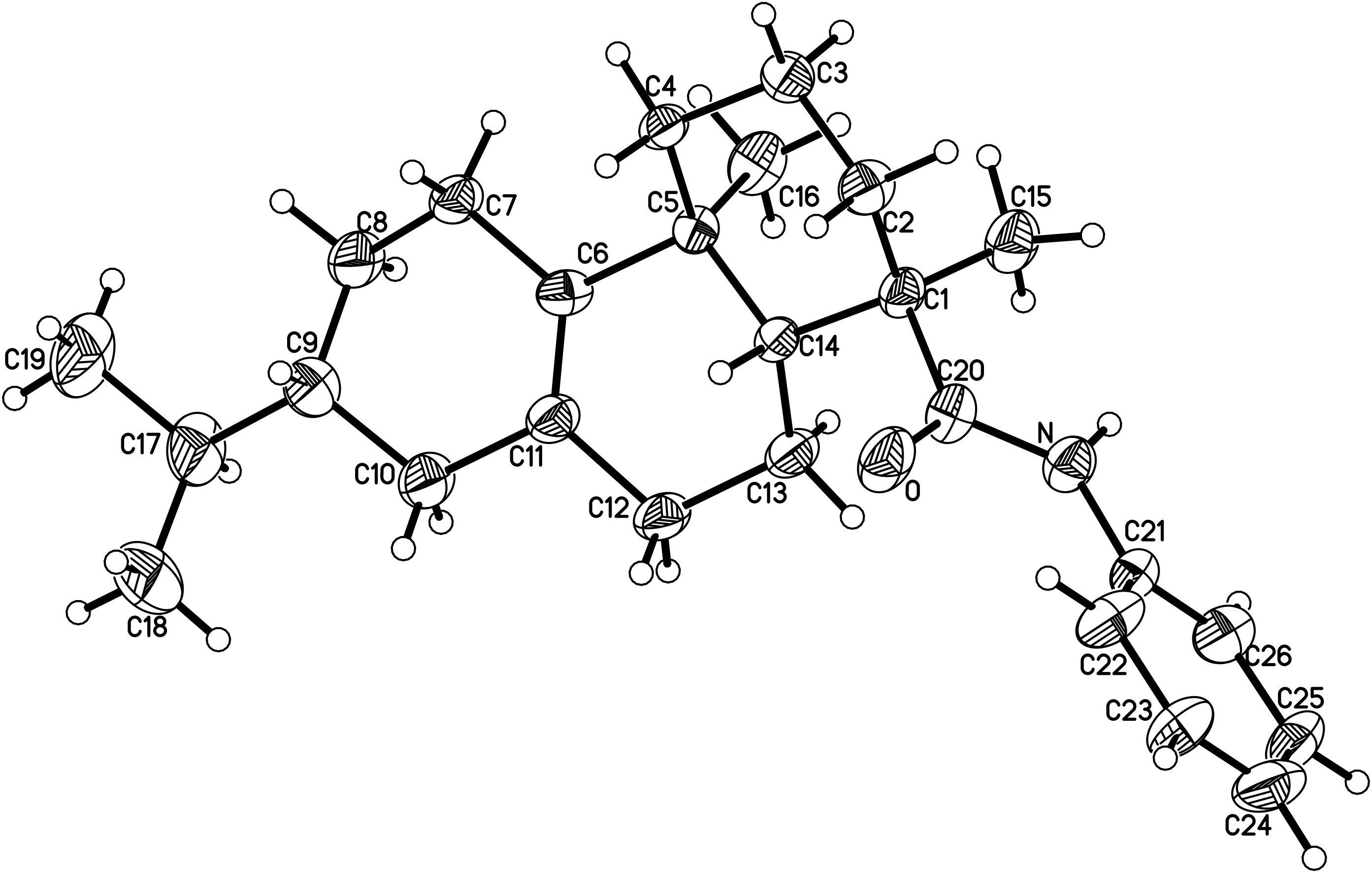

Supplement: Supplementary file 3 [file e-71-0o801-fig1.tif]
